# *Lactobacillus rhamnosus* Ameliorates Dyslipidemia and Liver Steatosis in a Rat Model Fed High-Fat Diet

**DOI:** 10.1155/grp/5540686

**Published:** 2025-10-13

**Authors:** Yu-Tso Liao, John Huang, Been-Ren Lin, Jin-Tung Liang, Kai-Wen Huang

**Affiliations:** ^1^Graduate Institute of Clinical Medicine, College of Medicine, National Taiwan University, Taipei, Taiwan (ROC); ^2^Division of Colorectal Surgery, Department of Surgery, National Taiwan University Hospital, Hsin-Chu Branch, Hsinchu, Taiwan; ^3^Division of Colorectal Surgery, Department of Surgery, National Taiwan University Hospital and College of Medicine, Taipei, Taiwan; ^4^Department of Surgery, National Taiwan University Hospital, Taipei, Taiwan; ^5^Hepatitis Research Centre, National Taiwan University Hospital, Taipei, Taiwan; ^6^Center for Functional Image and Interventional Therapy, National Taiwan University, Taipei, Taiwan

**Keywords:** *Lactobacillus rhamnosus* TCELL, metabolome, microbiota, nonalcoholic fatty liver disease

## Abstract

**Background:**

Nonalcoholic fatty liver disease (NAFLD) progression may be associated with dysbiosis of gut microbiota. Therefore, probiotics that can modulate gut dysbiosis are a potential strategy to reverse NAFLD. The aim of this study was to evaluate the protective effect of *Lactobacillus rhamnosus* TCELL (TCELL), a strain of probiotics *Lactobacillus rhamnosus*, on a rat model fed a high-fat diet (HFD).

**Materials and Methods:**

We used a rat model fed an HFD for 3 weeks. TCELL (10^9^ colony-forming units/day) were administered orally at the end of the third week for another 3 weeks. Body weights were measured, and serum, stool, and liver tissues were collected and analyzed after the animals were euthanized at the end of the sixth week. Serum samples were examined for lipid and metabolome profiles. Stool samples were examined for changes in the gut microbiota composition. Liver samples were examined to assess the lipid content.

**Results:**

After TCELL supplementation, the body weight and serum cholesterol levels decreased (*p* = 0.0088 and *p* = 0.0286, respectively). Additionally, the serum high-density lipoprotein/low-density lipoprotein ratio increased (*p* = 0.0177), and triglyceride lipid droplets decreased in the liver tissue (*p* = 0.0054). At the phylum level, TCELL supplementation increased the abundance of Firmicutes and the Firmicutes/Bacteroides ratio. At the species level, TCELL supplementation increased the abundance of *Allobaculum stercoricanis*, *Blautia glucerasea*, *Erysipelatoclostridium ramosum*, *Lactobacillus johnsonii*, *Blautia schinkii*, and *Anaerostipes caccae.* Based on the metabolome assay, the levels of acetic, propionic, butyric, and pentanoic acids decreased after feeding with HFD, and supplementation of TCELL significantly increased the level of isovaleric acid compared with that in the HFD group (*p* = 0.0418).

**Conclusions:**

TCELL supplementation decreased body weight gain, reversed serum lipid profiles, and lowered triglyceride lipid droplets in the liver tissue in a rat model. The beneficial effects of TCELL may be associated with the modulation of the gut microbiota composition and SCFA profiles.

## 1. Introduction

Nonalcoholic fatty liver disease (NAFLD) has gradually replaced viral hepatitis as the main cause of chronic liver disease, becoming a global burden for adults in recent decades [1]. NAFLD is caused by the reduced clearance of hepatic triglycerides or fatty acids due to excessive carbohydrate and lipid intake, obesity, and sedentary behavior and, consequently, increased accumulation of lipids in hepatocytes [2, 3]. If left untreated, NAFLD may lead to liver fibrosis, cirrhosis, and even hepatocellular carcinoma [2, 4]. The treatment of NAFLD mainly relies on physical exercise and abstinence from excessive carbohydrate and lipid intake [5]. Effective medicines for preventing or treating NAFLD are still under exploration [6].

The concept of the gut–liver axis emphasizes the role of gut microbiota in the pathophysiology of many liver diseases [7]. Gut microbiota can regulate energy homeostasis by means of increasing carbohydrate fermentation into short-chain fatty acids (SCFAs), thereby driving the resynthesis of triglycerides and bacterial-derived toxins in the liver [8]. Dysbiosis of gut microbiota causes disruption of gut integrity, leading to pathological conditions, including leaky gut and endotoxemia, which in turn contribute to low-grade inflammation and oxidative stress [9]. Recently, the relationship between dysbiosis of gut microbiota and the pathogenesis of NAFLD has been gradually acknowledged [10–12].

Probiotics are living organisms that confer benefits to human health [13]. Reports have shown in animal models that supplementation of probiotics can restore gut barrier function [14], immune function, and brain health [15]. Restoration of the gut microbiota by supplementation of probiotics may demonstrate similar beneficial effects on the reversal of the progress of human NAFLD; recent studies using animal models have reported that probiotic supplements readjusted the composition of gut microbiota and enhanced carbohydrate and lipid metabolism, thereby alleviating hepatic steatosis [16–18].

*Lactobacillus rhamnosus* is a species of commensal gut bacteria found in the human body and has been recognized by the academic community as a kind of probiotics beneficial for human health [19]. For example, *Lactobacillus rhamnosus* GG, a well-known subspecies of *Lactobacillus rhamnosus,* can elicit protective effects against dyslipidemia in animal models. These effects are mediated by restoring gut barrier function, antioxidant and anti-inflammatory properties [20], and modulation of gut microbiota composition [21], consequently ameliorating dyslipidemia caused by a high-fat diet (HFD) [21]. Further, *Lactobacillus rhamnosus* TCELL (TCELL) is a strain of *Lactobacillus rhamnosus* with specific sequencing and ribotyping [22] and was isolated from the duodenal and rectal mucosa of healthy residents in Taiwan. However, the protective effects of TCELL in animal models have not been elucidated in previous studies. In this study, we hypothesized that TCELL would exhibit a protective effect similar to that of other strains of *Lactobacillus rhamnosus by* reversing dyslipidemia in a rat model that was fed with HFD. We also investigated alterations in the gut microbiota and SCFA profiles in the rat model after supplementation of TCELL.

## 2. Results

### 2.1. Analysis of Body Weight, Serum Lipid Profile, and Liver Steatosis

**Figure 1:**
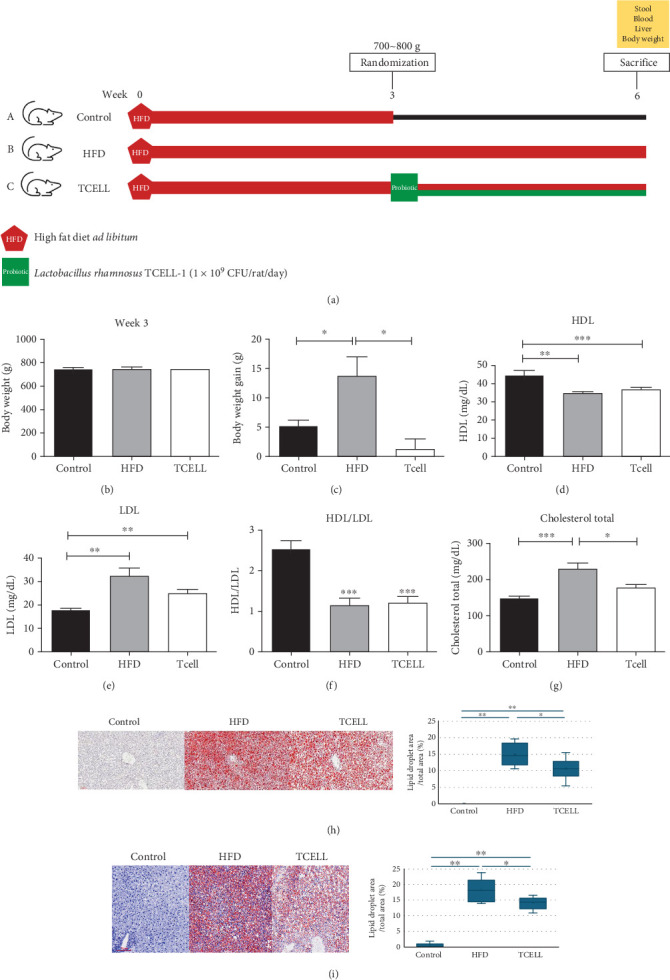
The clinical manifestations and lipid profiles induced by FHD can be ameliorated by TCELL supplement. (a) Experiment design. (b) Body weight of three groups in Week 3. (c) Body weight gain. (d) HDL. (e) LDL. (f) HDL/LDL. (g) Total cholesterol. (h) Representative images of liver tissue sections stained with H&E and lipid droplet density for control, HFD, and TCELL groups. (i) Representative images of liver tissue sections stained with Oil Red O and lipid droplet density among three groups. ⁣^∗^*p* < 0.05 and ⁣^∗∗^*p* < 0.005.

After randomization (Week 3), we measured the weight of rats in every group (Figure 1). The findings are listed as follows: 744.8 ± 30.4 (*n* = 10), 730 ± 95.0 (*n* = 10), and 737.5 ± 35.3 (*n* = 10) (grams) (Figure 1). After treatment under different conditions for another 3 weeks (Week 6), the weights of rats in every group were as follows: 749.8 ± 29.7, 736.6 ± 98.7, and 744.7 ± 36.0 (grams). We found that body weight gain was significantly reduced in the TCELL group compared to the HFD group (control group vs. HFD group, *p* = 0.0456; HFD group vs. TCELL group, *p* = 0.0088, separately) (Figure 1). The supplementation with TCELL also led to a decrease in the serum total cholesterol (control group vs. HFD group, *p* = 0.0012; HFD group vs. TCELL group, *p* = 0.0286, separately) and an increase in the serum high-density lipoprotein (HDL) levels and HDL/LDL ratio (*p* = 0.0177) (Figures 1, 1, 1, and 1).

Figure 1h,i shows the hematoxylin and eosin (H&E) and Oil Red O staining of rat liver slices. Rats in the HFD and TCELL groups showed an apparent increase in intrahepatic lipid accumulation compared with that observed for the control group. However, the supplementation of TCELL significantly decreased the number of lipid droplets induced by HFD on H&E and Oil Red O staining (*p* = 0.0124 and *p* = 0.0054, respectively).

### 2.2. Characterization of Bacterial Diversity and Composition

**Figure 2:**
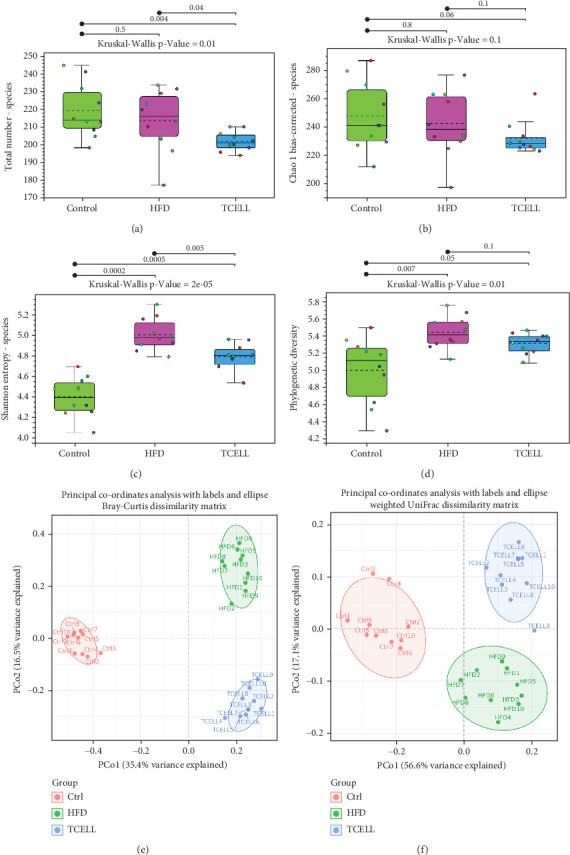
Alpha and beta diversity of gut microbiota in three groups. (a) Boxplots of observed OTUs (≥ 97% identity level). (b) Chao1 estimator of richness. (c) Shannon index. (d) Phylogenetic diversity of 30 fecal samples from three groups. (e) Principal coordinate analysis (PCoA) plots obtained from sequencing the microbiota in fecal samples. PCoA according to the Bray–Curtis index. (f) PCoA according to weighted UniFrac analysis.

Alpha and beta diversities were evaluated for the three groups (Figure 2). For alpha diversity, the richness of the gut microbiota was evaluated based on the observed total numbers and Chao1 estimator, showing that the HFD group lost alpha diversity of the gut microbiota compared to the control group. Supplementation of TCELL further decreased the alpha diversity compared to that observed for the HFD group (Figure 2a,b). Richness and evenness were then evaluated based on the Shannon index and phylogenetic diversity. We observed that the HFD group exhibited higher richness and evenness compared with the control group; however, the TCELL group had lower richness and evenness compared with the HFD group (Figure 2c,d).

Beta diversity among the three groups was evaluated using the Bray–Curtis index and weighted UniFrac (unique fraction) analysis. Principal coordinate analysis (PCoA) plots based on these two methods showed that the clusters of gut microbiota in the three groups were clearly separated from each other (Figure 2e,f).

### 2.3. Changes in the Taxonomic Profiles of Gut Bacteria

**Figure 3:**
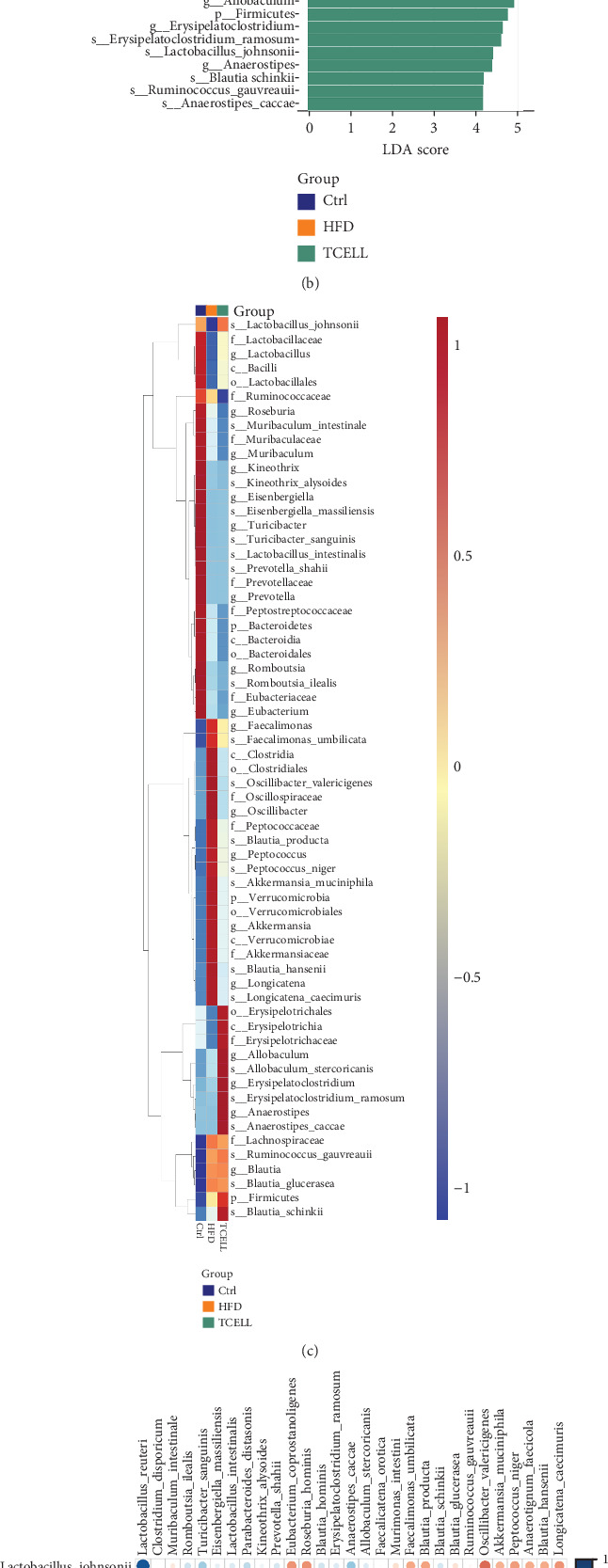
Linear discriminant analysis (LDA) effect size (LEfSe) results on gut microbiota in three groups. (a) Cladogram depicts the evolutionary relationship between bacterial units that are differentially abundant between three groups. (b) Histogram represents that the LDA scores of bacteria with significant differential abundance between three groups. The threshold of log LDA score was set as 4. (c) Heatmap indicates the abundance of gut microbiota in three groups. (d) Spearman correlation analysis between the top 30 species in all samples. (e) Spearman correlation network analysis between the top 30 species in all samples. Each circle indicates a single species. The green line represents positive correlation and the red line represents negative correlation. Line thickness denotes the strength of correlation. The correlation coefficient is set as 0.7.

The taxonomic profiles of the gut microbiota in each group were analyzed at different levels. To compare the statistical and biological taxonomic differences between the gut microbiota in the three groups, linear discriminant analysis (LDA) effect size (LEfSe) algorithm with an LDA score cutoff≧4 was used. This analysis identified 56 taxa (Figure 3).

**Figure 4:**
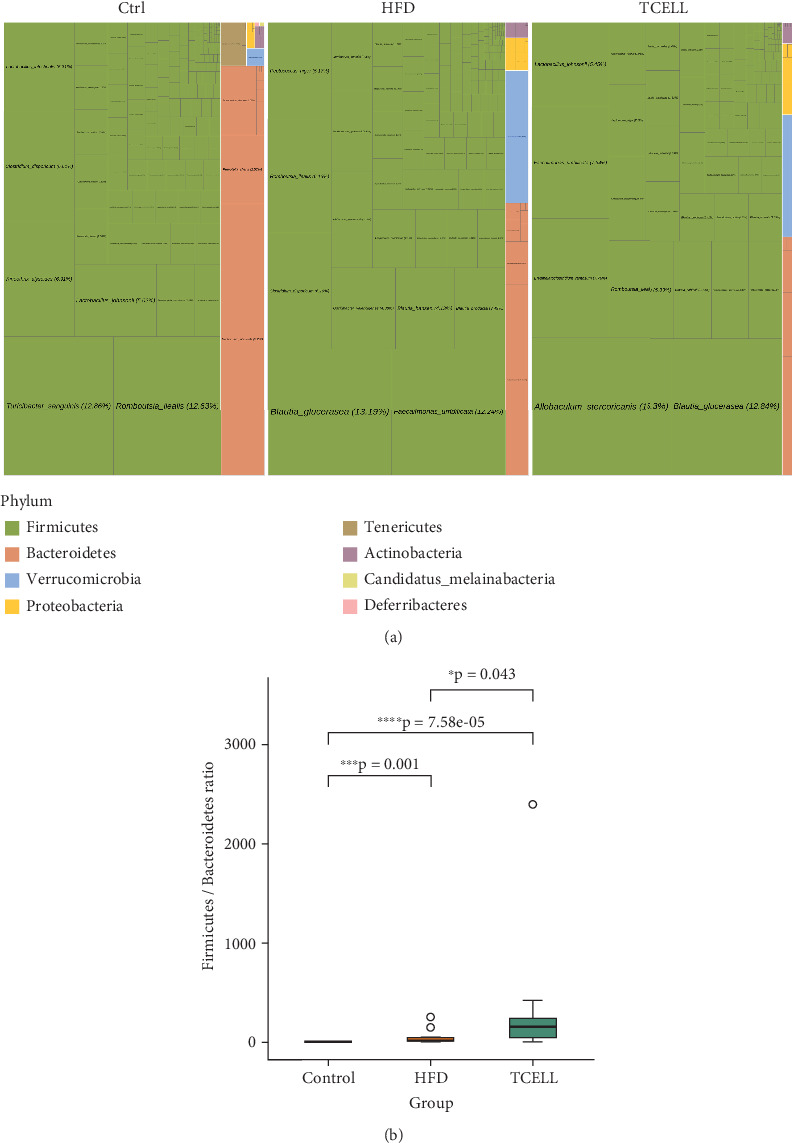
Taxonomic profiles of the fecal bacteria at phylum level in three groups. (a) The tree map shows that the Firmicutes/Bacteroides ratio increases in the HFD and TCELL group. (b) Firmicutes/Bacteroides ratio in three groups.

At the phylum level, the tree map showed that phyla Firmicutes and Bacteroides were dominant in all samples. The percentages of Firmicutes and Bacteroides increased in the HFD group and further increased in the HFD/TCELL group (Figure 4a). When considering the Firmicutes/Bacteroides ratio, we found that this ratio increased in the HFD group and further increased in the HFD/TCELL group (Figure 4b).

**Figure 5:**
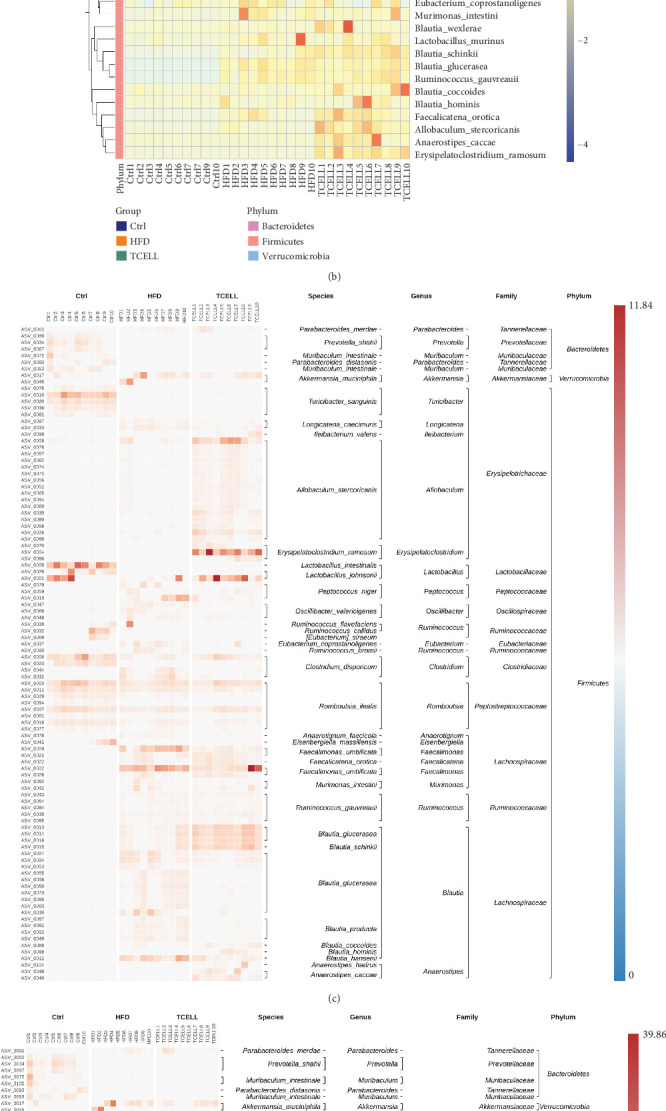
Composition of gut microbiota in three groups. (a) Values represent the relative abundance of the top 10 species. (b) Heatmap analysis of the bacterial distribution among the 30 samples based on hierarchical clustering. (c) Clustered heatmap represents relative abundance of top 100 species (*n* = 10 per group). (d) Cluster heatmap represents relative abundance of top 100 species. In this cluster heatmap, the relative abundance of single bacterium is normalized by *Z*-score standardization for all samples.

At the genus level, the HFD group had an increased abundance of *Lachnospiraceae*, *Faecalimonas*, *Peptococcus*, *Oscillospiraceae*, and *Oscillibacter*, whereas supplementation of *Lactobacillus rhamnosus TCELL-1* increased the abundance of *Blautia*, *Allobaculum*, *Erysipelatoclostridium*, and *Anaerostipes* (Figure 5). At the species level, the HFD group showed an increased abundance of *Faecalimonas umbilicate*, *Peptococcus niger*, *Blautia hansenii*, *Blautia producta*, *Oscillibacter valericigenes*, and *Ruminococcus gauvreauii*, whereas supplementation of TCELL resulted in increased abundance of *Allobaculum stercoricanis*, *Blautia glucerasea*, *Erysipelatoclostridium ramosum*, *Lactobacillus johnsonii*, *Blautia schinkii*, and *Anaerostipes caccae* (Figure 5).

### 2.4. Changes in the SCFA Profiles

**Figure 6:**
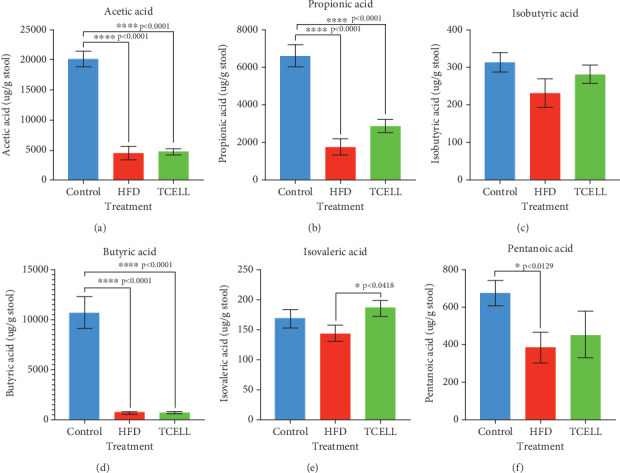
(a–f) Changes of SCFA profiles in three groups. The level of all six SCFAs increase, but only the level of isovaleric acid increases with statistical significance after 3 weeks of TCELL supplementation (*p* = 0.0418).

Six metabolites were measured in stool samples (Figure 6). We found that the levels of acetic acid, propionic acid, butyric acid, and pentanoic acid were significantly lower in the HFD group than those in the control group (Figures 6a, 6b, 6d, and 6f). Furthermore, the levels of all SCFAs, including acetic acid, propionic acid, isobutyric acid, butyric acid, isovaleric acid, and pentanoic acid, increased after the supplement of TCELL (Figures 6a, 6b, 6c, 6d, 6e, and 6f), though only isovaleric acid levels increased with statistical significance after 3 weeks of TCELL supplementation (*p* = 0.0418) (Figure 6e).

## 3. Discussion

Here, we demonstrated the safety of the oral supplementation with the probiotic TCELL, a strain of *Lactobacillus rhamnosus* isolated from the duodenal and rectal mucosa of healthy residents in Taiwan in an HFD-treated rat model. Supplementation with TCELL reduced body weight gain, reversed serum lipid profiles, and lowered triglyceride lipid droplets in liver tissue. Sequential taxonomic and SCFA alterations caused by HFD and oral supplementation with TCELL were observed. Taken together, our study indicates a protective role of TCELL in ameliorating liver steatosis and injury in an HFD-treated rat model. These changes may be associated with the modulation of gut microbiota composition and SCFA profiles.

First, in line with other strains of *Lactobacillus rhamnosus*, TCELL exhibited similar protective effects in rodent models fed an HFD [20, 21, 23–26]. For example, *Lactobacillus rhamnosus* GG, a well-known probiotic, inhibits body weight gain and decreases the levels of serum lipids, such as cholesterol and triglycerides, in an HFD-induced mouse model [20, 23]. Furthermore, a supplement of *Lactobacillus rhamnosus* GG reversed liver steatosis in this mouse model [24]. Similar protective effects have been observed with other strains of *Lactobacillus rhamnosus*, including L7-1, L101, and LA68 [25, 26]. Our study confirms that TCELL, a new strain of *Lactobacillus rhamnosus*, exhibits effects similar to those of other strains of *Lactobacillus rhamnosus* in a rat model fed an HFD. The findings warrant further investigation [20, 23–26].

In contrast to previous studies, we evaluated the effect of TCELL on rats with pre-existing steatosis and liver injury induced by HFD treatment for 3 weeks. Indeed, most previous studies have evaluated the effect of probiotics using healthy rodent models fed either HFD or HFD plus probiotics [20, 21, 24, 27]. Baseline serum liver enzyme levels, lipid profiles, and liver steatosis differed between healthy rats and those fed HFD for 3 weeks, along with gut microbiota and their metabolomes. Therefore, the protective effects of *Lactobacillus rhamnosus* against fatty liver disease in rodent models should be investigated separately. *Lactobacillus rhamnosus* TCELL retained beneficial effects in rats that were pretreated with HFD for 3 weeks, a scenario applicable to most individuals with pre-existing fatty liver disease initiating probiotic supplementation.

To understand the underlying taxonomic changes induced by HFD and *Lactobacillus rhamnosus* TCELL, the composition of the gut microbiota was analyzed and compared among the three groups using metagenomics of 16s rRNA whole genome sequencing. The results showed that HFD decreased alpha diversity. Supplementation with *Lactobacillus rhamnosus* TCELL further decreased the alpha diversity. Supplement of TCELL reducing alpha diversity is biologically counterintuitive for a probiotic. To explain the findings in our study, we postulated that the ability of single-strain probiotics of *Lactobacillus rhamnosus* to increase abundance of gut diversity may be limited. Wang et al. reported that concomitant administration of *Lactobacillus rhamnosus* GG to rats fed with HFD for 23 weeks did not significantly increase alpha diversity [26]. Similar findings were also reported by Geng et al. [17] and Jang et al. [24] that alpha diversity did not significantly increase in rats fed with both HFD and *Lactobacillus rhamnosus* GG for 8 and 9 weeks, separately. Together with the findings in our study, we suggest that the association between alpha diversity and single strain of *Lactobacillus* supplementation should be clarified in future studies. Our study evaluated the beta diversity of the three groups using PCoA. The HFD changed the microbiota composition. Furthermore, the supplementation altered microbiota composition in the control and HFD groups. At the phylum level, the HFD group showed elevated Firmicutes abundance and Firmicutes/Bacteroides ratio. The elevated Firmicutes/Bacteroides ratio is associated with obesity. Obese patients demonstrate an increased Firmicutes/Bacteroides ratio compared to lean patients [28]. Additionally, we showed that supplementation with TCELL in the HFD group led to higher Firmicutes abundance and Firmicutes/Bacteroides ratio than those observed in the HFD-only group. We suggested that TCELL belongs to the Firmicutes phylum. Hence, short-term supplementation of TCELL may lead to an increase in Firmicutes abundance and Firmicutes/Bacteroides ratio. Many previous studies have reported similar findings in rodent models supplemented with *Lactobacillus* [18, 21, 24]. We hypothesize that the long-term supplementation with TCELL may lower the Firmicutes/Bacteroides ratio. As reported by Geng et al., the Firmicutes abundance decreased and Bacteroides abundance increased, together leading to a decreased Firmicutes/Bacteroides ratio after 8 weeks of *Lactobacillus rhamnosus* GG supplementation [17]. Also reported by Kim et al., the Firmicutes/Bacteroides ratio decreased after being fed with *Lactobacillus rhamnosus GG* for 13 weeks [23]. Because TCELL and *Lactobacillus rhamnosus GG* belong to the same species *Lactobacillus rhamnosus*, we therefore hypothesize that the long-term supplementation with TCELL may also lower the Firmicutes/Bacteroides ratio as the effect that *Lactobacillus rhamnosus* GG exhibits. However, further studies are needed to confirm this hypothesis.

Supplementation with TCELL altered the microbiota composition modulated by HFD. Furthermore, it increased the abundance of *Blautia*, *Allobaculum*, *Erysipelatoclostridium*, and *Anaerostipes*. Increased abundance of the two species *Blautia glucerasea* and *Blautia glucerasea* was observed upon TCELL supplementation. The genus *Blautia* exerts beneficial effects on glucose metabolism and can decrease obesity-associated inflammation [29]. Our findings suggest that an increase in the abundance of beneficial commensal gut bacteria may be one of the mechanisms by which TCELL attenuates the severity of weight gain, dyslipidemia, and liver steatosis.

At the species level, the abundance of *Lactobacillus johnsonii* increased after TCELL supplementation. *Lactobacillus johnsonii* ameliorates diet-related dyslipidemia [30]. In addition, the abundance of *Anaerostipes caccae* increased after TCELL supplementation*. Anaerostipes caccae* is a bacterial strain capable of producing butyric acid [31], is abundant in human infant feces [31], and is capable of preventing allergies [32]. The beneficial effects of the *Lactobacillus* genus in humans have been postulated to be associated with many mechanisms [33]. One of these factors might be related to an increase in the abundance of other commensal microbiota [34]. Our study suggests that the supplementation of *Lactobacillus* can increase the number of commensal bacteria, which may have the potential to attenuate the severity of fatty liver disease in a rat model.

Changes in the concentrations of the six SCFAs were measured to elucidate changes in the metabolome after HFD and TCELL supplementation. The levels of acetic, propionic, butyric, and pentanoic acids decreased after the HFD treatment, consistent with a previous study [26]. These SCFAs serve as energy resources for colonocytes, enhance the integrity of the gut barrier, and regulate host immunity [35]. SCFAs, including acetic, propionic, and butyric acids, are products of undigested fibers fermented by the gut microbiota [35]. Furthermore, acetic and propionic acids are formed by bacteria belonging to the phyla Bacteroides and Firmicutes [36, 37]. Accordingly, HFD-induced depletion of bacteria belonging to the phylum Bacteroides may explain the findings of our study. Moreover, after 3 weeks of TCELL supplement, the levels of six SCFAs increased, including acetic acid, propionic acid, isobutyric acid, butyric acid, isovaleric acid, and pentanoic acid; however, only isovaleric acid levels increased with statistical significance (*p* = 0.0418) (Figure 6e). A similar finding of fecal elevation of isovaleric acid has also been observed in children supplemented with *Lactobacillus paracasei* Lpc-37 [38]. Butyric acid is a product produced by *Lactobacillus* [21]. Although butyric acid did not increase with statistical significance in our study, we postulate butyric acid will increase significantly after a longer time of TCELL supplement, as exhibited in previous studies [21, 39]. Notably, the acetic acid is the most common and sensitive SCFA regulated by beneficial bacteria [40]. However, in our study, there was no significant change in acetic acid concentrations after TCELL supplement. This finding may be associated with two factors. First, it may be associated with the ability and the duration to produce acetic acid by TCELL. As reported by Wang et al., acetic acid did not increase after 23 weeks of *Lactobacillus rhamnosus* GG supplement in rats fed with HFD diet [26]. It may be that probiotics do not exert significant effects after only 3 weeks even though we insist on gavage every day; however, this question cannot be answered in our study and requires future study to clarify. Second, while reviewing the data for acetic acid level, we found the majority of the acetic acid in TCELL group increased except for three samples (No. 2, No. 6, and No. 10). We hypothesized that the individual differences toward TCELL may affect the findings in our study in the short-term supplement of TCELL, despite the trend of increasing acetic acids persisting. We thought a longer TCELL supplement may be needed to clarify the impact of TCELL on acetic acid level in future study.

Some unresolved questions from our study need to be addressed. First, the findings only showed an association between the beneficial effects of TCELL in a rat model and alterations in the microbiota taxonomy and SCFAs. However, the exact mechanism underlying the amelioration of body weight loss, dyslipidemia, and liver steatosis by TCELL remains unknown. For example, one of the mechanisms underlying the protective effects of *Lactobacillus* is associated with an increased abundance of beneficial commensal bacteria [24, 34]; however, this effect was not observed in our study. Future studies are needed to determine the direct and/or indirect effects of TCELL as well as those of commensal beneficial bacteria. Second, the abundance of *Blautia*, a genus of gut microbiota that can produce butyrate, was significantly increased after TCELL supplementation. Although the butyric acid level did increase, no significant change in butyric acid levels was observed in our study. Besides butyric acid, acetic acid is the most abundant SCFA and the most responsive to regulation by beneficiary bacteria; however, its levels did not increase in this study. This may be attributed to the short-term supplement of TCELL in rats fed with HDF for 3 weeks. It is plausible that probiotics do not legally colonize in the gut and exert effects after only 3 weeks. A longer period of TCELL supplementation should be employed in future studies. The levels of SCFA should be monitored in future studies. In addition, validation studies using biochemical markers are required. Third, in this study, the rats were fed an HFD only for 6 weeks. Generally, to build nonalcoholic fatty liver in a short period of time is difficult by adopting a high-sugar diet based on fructose. Nonetheless, the H&E and Oil Red O staining indicated large lipid deposition in the rat liver, which has been confirmed as nonalcoholic fatty liver. The methionine- and choline-deficient (MCD) diet is the best-established model for studying NAFLD in rat models in a short time. Methionine, an essential sulfur-containing amino acid, plays a role in hepatic lipid metabolism. A methionine-deficient diet is a well-established model for studying NAFLD [41]. Reviewing the diet ingredients in our study (Research Diets, Catalog No. D0910031i), we found that this diet formula lacks methionine. Therefore, we suggest that a methionine-deficient HFD is one of the factors that can construct an HFD model in 6 weeks.

In conclusion, TCELL, a newly isolated strain of *Lactobacillus rhamnosus*, ameliorated HFD-induced dyslipidemia and liver steatosis in a rat model. The beneficial effects of TCELL may be associated with the modulation of the gut microbiota composition and SCFA profiles. TCELL exhibited similar beneficial effects to *Lactobacillus rhamnosus* in a rat model fed an HFD and warrants further investigation.

## 4. Materials and Methods

### 4.1. Probiotic Strains

TCELL is a strain of *Lactobacillus rhamnosus*, which was isolated from the duodenal and rectal mucosa of healthy residents in Taiwan. The TCELL strain was purchased from T-CELL Biotechnology Food Co. Ltd. and stored at 4°C until further use.

### 4.2. Animal Model

Eight-week-old male Wistar rats were purchased from the National Laboratory Animal Center (Taipei, Taiwan). Thirty rats were housed in cages at 23°C ± 2°C and 55% ± 5% relative humidity under a 12-h light/dark cycle. After acclimatization for 1 week, the rats were provided ad libitum access to HFD (Research Diets, Catalog No. D0910031i, containing 40 kcal% fructose, 20 kcal% fructose, and 2% cholesterol) for 3 weeks. The duration of HFD administration was based on previous studies [23–25]. Each rat was weighed after being fed with HFD for 3 weeks. Animals were randomized into three groups: control, HFD, and HFD plus TCELL (TCELL). The rats in each group were weighed again to ensure comparability among the three groups. The rats in the control group were not fed HFD after randomization. The rats in the HFD group were fed HFD for 3 weeks after randomization until sacrifice. The rats in the TCELL group were fed an HFD along with TCELL at a dosage of 1 × 10^9^ CFU/day after randomization. The TCELL group was used to evaluate the protective effect of TCELL against HFD-induced fatty liver in rats. Blood and stool samples were collected in Weeks 3 and 6 after the initiation of the experiment. All rats were euthanized with 1% pentobarbital sodium solution by intraperitoneal injection. Liver tissues were collected and fixed in 4% neutral formalin for further analysis (Figure 1a).

All animal procedures were approved by the Institutional Animal Care Committee of the National Taiwan University Hospital, and the animals were maintained in accordance with the guidelines of the Animal Experimental Center of the National Taiwan University Hospital (Approval No. 20201195).

### 4.3. Biochemical Analysis

Serum levels of alanine aminotransferase (ALT), aspartate aminotransferase (AST), triglycerides, total cholesterol, LDL, and HDL were measured. Body weights were measured at Weeks 0, 3, and 6.

### 4.4. 16S RNA Gene Sequencing

To ensure data reliability, quality control was performed at each step of the study (i.e., DNA sampling, PCR testing, library preparation, and sequencing). Total fecal genomic DNA was extracted using a column-based method with a QIAamp PowerFecal DNA Kit (Qiagen). DNA concentration was determined by Qubit 4.0 Fluorometer (Thermo Scientific) and adjusted to 1 ng/*μ*L for the subsequent procedures.

Full-length 16S rRNA gene (V1–V9 regions) was amplified using barcoded 16S gene-specific primers. According to the Amplification of Full-Length 16S Gene with Barcoded Primers for Multiplexed SMRTbell Library Preparation and Sequencing Procedure (PacBio), each primer was designed to include a 5⁣′ buffer sequence (GCATC) with a 5⁣′ phosphate modification, a 16-base barcode, and the degenerate 16S gene-specific forward or reverse primer sequences (forward: 5⁣′-Phos/GCATC-16-base barcode-AGRGTTYGATYMTGGCTCAG-3⁣′, reverse: 5⁣′-Phos/GCATC-16-base barcode-RGYTACCTTGTTACGACTT-3⁣′). Degenerate base identities were as follows: R = A, G; Y = C, T; M = A, C. Briefly, 2 ng of gDNA was used for the PCR reaction carried out with KAPA HiFi HotStart ReadyMix (Roche) under the following PCR conditions: 95°C for 3 min; 20~27 cycles (sample dependence) of 95°C for 30 s, 57°C for 30 s, 72°C for 60 s; 72°C for 5 min; and hold at 4°C. The PCR products were assessed using 1% agarose gel. Samples with a bright main strip of approximately 1500 bp were chosen and purified using AMPure PB Beads for subsequent library preparation.

The SMRTbell library was prepared by amplifying the full-length 16S gene using Barcoded Primers for Multiplexed SMRTbell Library Preparation and Sequencing Procedure (PacBio). In brief, equal molar amounts of each barcoded PCR product were pooled and 500–1000 ng of pooled amplicon sample was used for DNA damage repair, followed by end-repair/A-tailing and ligation steps to introduce the universal hairpin adapters onto double-stranded DNA fragments. After purification with AMPure PB beads to remove the adapter dimer, the SMRTbell library was incubated with sequencing primer v4 and Sequel II Binding Kit 2.1 for primer annealing and polymerase binding. Finally, sequencing was performed in circular consensus sequence (CCS) mode on a PacBio Sequel IIe instrument to generate HiFi reads with a predicted accuracy (Phred Scale) of 30.

The CCS reads were determined with a minimum predicted accuracy of 0.9, and the minimum number of passes was set to three in the official workflow of PacBio using SMRT Link software. After demultiplexing, the CCS reads were further processed using DADA2 (Version 1.20) to obtain amplicons with single-nucleotide resolution [42]. The DADA2 workflow includes quality filtering, dereplication, learning of the dataset-specific error model, amplicon sequence variant (ASV) inference, and chimera removal. Trimming and filtering were performed with a maximum of two expected errors per read (maxEE = 2). The DADA2 algorithm resolves exact ASVs with single-nucleotide resolution from the full-length 16S rRNA gene, with a near-zero error rate. For each representative sequence, the feature-classifier [43] and classify-consensus-vsearch [44] algorithms in QIIME2 [45] were employed to annotate the taxonomic classification based on the information retrieved from the NCBI database. To analyze the sequence similarities among different ASVs, multiple sequence alignments were conducted using the QIIME2 alignment MAFFT [46] against the NCBI database [47].

### 4.5. Histological Analysis

Liver tissues were fixed in 4% paraformaldehyde for 24 h. The fixed liver tissues were embedded in the Tissue Tek O.C.T., sectioned, and stained with H&E and Oil Red O. The stained tissues were analyzed by light microscopy.

### 4.6. SCFA Analysis

The fecal SCFAs were quantified using Agilent 7890A gas chromatograph (Agilent Technologies, Santa Clara, California) and Pegasus 4D GC × GC-TOFMS system (Leco Corporation, St. Joseph, Michigan, United States). First, 20-mg feces were diluted with 500 *μ*L 0.5% H_3_PO_4_ aqueous solution. The diluted samples were homogenized with Geno/Grinder at 1000 rpm for 2 min and centrifuged at 18,000 rcf for 10 min at 4°C. Next, we collected 285-*μ*L mixed sample solution and then added 15-*μ*L acetate-d3 into the mixed sample solution. Then, 300-*μ*L butanol was added for the liquid–liquid extraction of SCFAs. Again, we homogenized and centrifuged the mixture. The 20-*μ*L internal standard propionate-d5 was added into 180-*μ*L upper organic layer. Finally, we filtered and transferred to the GC-TOFMS system for analysis.

### 4.7. Statistical Analysis

Descriptive statistics were used for all the data. The Wilcoxon test, Kruskal–Wallis test, and Dunn's post hoc analysis were used for continuous variables.

For analyzing the differences in gut microbiota among the three groups, the observed total numbers and Chao1 estimator were used to evaluate alpha diversity. These two indices were used to determine gut microbiota richness. The Shannon index and phylogenetic diversity were used to evaluate the richness and evenness of the gut microbiota.

Beta diversity was evaluated using Bray–Curtis dissimilarity and unweighted UniFrac distances. Bray–Curtis dissimilarity represents between-group similarity based on the gut microbiota composition (OTUs). UniFrac measures between-group similarity by calculating UniFrac distances, a phylogenetics-based distance metric.

The LEfSe algorithm was used to compare the statistical and biological taxonomic differences between the gut microbiota of the three groups. This method considered OTU differences in terms of both abundance and frequency among the three groups. The nonparametric Kruskal–Wallis test and LDA were utilized to examine the statistical significance, and an LDA score ≥ 4 was considered significant. Spearman's correlation was used to evaluate the association between the abundance of the gut microbiota. A Spearman correlation coefficient of 0.7 was set to indicate a strong positive monotonic relationship between two species. In the present study, the criteria for the top 30 species are based on abundance for the relative number of times a specific ASV appears in the specimens. The Kruskal–Wallis test was used to compare several different groups. The network analysis was used to evaluate and visualize the relationships between different species.

## Data Availability

The data that support the findings of this study are available from the corresponding author, upon reasonable request.
